# Supplementary material for: The dynamics of carbon emissions, energy, income, and life expectancy: Regional comparative analysis
Source: PLoS One. 2024 Feb 22;19(2):e0293451. doi: 10.1371/journal.pone.0293451 (PMC10883542; doi:10.1371/journal.pone.0293451)
Supplement: S1 Table — (DOCX) [file pone.0293451.s002.docx]

**Dickey-fuller ( levels)**

| **variables** | **Carbon emissions** | **GDP per capita** | **Life expectancy** | **Death rate** | **Energy** | **population** |
| --- | --- | --- | --- | --- | --- | --- |
| Inverse chi-squared(192) p | 2247.1387  (0.0000) | 138.0544  (0.9988) | 2632.1990  (0.0000) | 2710.6318  (0.0000) | 1308.4860  (0.0000) | 2531.5039  (0.0000) |
| Inverse normal Z | -41.1728  (0.0000) | 6.9098  (1.0000) | -45.1964  (0.0000) | -46.0722  (0.0000) | -28.5952  (0.0000) | -44.2830  (0.0000) |
| Inverse logit t(484) L* | -63.2796  (0.0000) | 7.1327  (1.0000) | -74.1314  (0.0000) | -76.3403  (0.0000) | -39.6281  (0.0000) | -71.2942  (0.0000) |
| Modified inv. chi-squared Pm | 104.8759  (0.0000) | -2.7529  (0.9970) | 124.5259  (0.0000) | 128.5284  (0.0000) | 63.1937  (0.0000) | 119.3873  (0.0000) |
| **Dickey-fuller unit root tests log forms with trend, with cross-sectional means and lag of 1** | | | | | | |
| variables | Carbon emissions | GDP per capita | Life expectancy | Death rate | Energy | population |
| Inverse chi-squared(192) p | 952.6133  (0.0000) | 450.0935  (0.0000) | 1047.5030  (0.0000) | 1148.5242  (0.0000) | 1034.8912  (0.0000) | 2203.5926  (0.0000) |
| Inverse normal Z | -21.6617  (0.0000) | -1.5329  (0.0000) | -24.0897  (0.0000) | -26.0648  (0.0000) | -23.4747  (0.0000) | -40.5003  (0.0000) |
| Inverse logit t(484) L* | -26.0548  (0.0000) | -5.2456  (0.0000) | -29.2217  (0.0000) | -32.1974  (0.0000) | -30.9291  (0.0000) | -62.0466  (0.0000) |
| Modified inv. chi-squared Pm | 38.8149  (0.0000) | 13.1708  (0.0000) | 43.6572  (0.0000) | 48.8124  (0.0000) | 48.0869  (0.0000) | 102.6537  (0.0000) |
