# Supplementary material for: The dynamics of carbon emissions, energy, income, and life expectancy: Regional comparative analysis
Source: PLoS One. 2024 Feb 22;19(2):e0293451. doi: 10.1371/journal.pone.0293451 (PMC10883542; doi:10.1371/journal.pone.0293451)
Supplement: Attachment — Submitted filename: PONE-comments.docx [file pone.0293451.s003.docx]

This manuscript report on “The dynamics of carbon emissions, energy, income, and life expectancy: Regional comparative analysis” The paper is rather perplexing as it deals with a familiar topic. Moreover, the study real-time contribution is also not clear. However, the reviewer proposes major revisions with the following recommendations:

1. The abstract needs to be rewritten to provide a clearer and more concise summary of the research. The current abstract is too technical and does not clearly communicate the main findings of the research.
2. The introduction is poorly structured and inadequately composed, and it should highlight the subject matter that the authors addressed as well as the empirical and theoretical contributions of the research.
3. The literature review section is badly structured and presented. You must redesign in such a way that it demonstrates how much literature fill problem you intend to solve and which literature gap you intend to fill via your study.
4. The authors need to provide a clearer justification for why they selected the specific variables used in their analysis.
5. The methodology section needs to be revised to provide a clearer description of the statistical methods used, including equations and any assumptions made.
6. The authors should consider including additional statistical tests or sensitivity analyses to test the robustness of their findings.
7. The methodology section needs to be revised to provide a clearer description of the statistical methods used, including equations and any assumptions made.
8. The methodology section needs to be revised to provide a clearer description of the statistical methods used, including equations and any assumptions made.
9. The authors should provide a more detailed discussion of the implications of their findings for policymakers, particularly as they relate to the Sustainable Development Goals.
10. Overall, the paper's concept is unique; nevertheless, non-academic language should be avoided in some cases, and in-text references should be proofed.
