# Supplementary material for: The dynamics of carbon emissions, energy, income, and life expectancy: Regional comparative analysis
Source: PLoS One. 2024 Feb 22;19(2):e0293451. doi: 10.1371/journal.pone.0293451 (PMC10883542; doi:10.1371/journal.pone.0293451)
Supplement: Attachment — Submitted filename: Response to reviewers.docx [file pone.0293451.s004.docx]

Response to reviewers

1. The abstract needs to be rewritten to provide a clearer and more concise summary of the research. The current abstract is too technical and does not clearly communicate the main findings of the research.

Response:

We appreciate the feedback provided regarding the clarity and conciseness of the abstract. We have carefully considered your suggestions and have revised the abstract to ensure a more accessible and concise summary of our research. The updated abstract now clearly communicates the main findings of our study in a manner that is understandable to a wider audience. Thank you for your valuable input, and we hope that the revised abstract meets the desired requirements.

2. The introduction is poorly structured and inadequately composed, and it should highlight the subject matter that the authors addressed as well as the empirical and theoretical contributions of the research.

Response:

We appreciate the feedback provided regarding the structure and composition of the introduction. We apologize for any shortcomings in clearly highlighting the subject matter and the empirical and theoretical contributions of our research. We understand the importance of effectively conveying these aspects to readers. In response to your valuable input, we have thoroughly revised the introduction to ensure it provides a clear overview of the subject matter addressed in our research. We have also emphasized the empirical and theoretical contributions, highlighting their significance in advancing the existing body of knowledge on the topic.

Thank you for bringing this to our attention, and we hope the revised work is an improvement over the previous work.

3. The literature review section is badly structured and presented. You must redesign in such a way that it demonstrates how much literature fill problem you intend to solve and which literature gap you intend to fill via your study.

Response:

We appreciate the feedback regarding the structure and presentation of the literature review section. We understand the importance of clearly demonstrating the existing literature's relevance to the problem we aim to address and highlight the specific gap our study intends to fill. In response to your valuable input, we have redesigned the literature review section to effectively showcase the depth and breadth of the literature pertaining to the problem at hand. We have emphasized the existing research's contributions, as well as identify the specific gap that our study seeks to fill. By doing so, we hope to have provided a comprehensive understanding of the research landscape and emphasized the significance of our study in addressing the identified gap.

4. The authors need to provide a clearer justification for why they selected the specific variables used in their analysis.

Response:

We appreciate the feedback regarding the justification for the selection of variables in our analysis. We understand the importance of providing a clear rationale for our variable choices to enhance the transparency and robustness of our research. In response to your valuable input, we have provided a more detailed and explicit justification for why we selected the specific variables used in our analysis. We have explained the theoretical underpinnings and empirical evidence supporting their relevance to the research question and objectives. As a consequence of these changes we hope to strengthen the methodological foundation of our study and provide a solid basis for the interpretation of our results.

5. The methodology section needs to be revised to provide a clearer description of the statistical methods used, including equations and any assumptions made.

Response:

We appreciate the feedback regarding the clarity of the methodology section, particularly in describing the statistical methods used, including equations and assumptions made. We recognize the significance of offering a detailed and thorough explanation of our methodology to ensure that our study is transparent and can be replicated by other researchers. In response to your valuable input, we have thoroughly revised the methodology section to provide a clearer and more detailed explanation of the statistical methods employed. The do files and the data used as well as their sources are provided in a repository for replication purposes. We have included relevant equations and provided a step-by-step description of the methodology used in our analysis. Additionally, we have explicitly outlined any assumptions made during the analysis to ensure transparency in our approach. By making these improvements, we aim to enhance the clarity and understanding of our methodology, enabling readers to replicate our study and assess the robustness of our findings.

6. The authors should consider including additional statistical tests or sensitivity analyses to test the robustness of their findings.

Response:

We appreciate the suggestion to include additional statistical tests or sensitivity analyses to further test the robustness of our findings. We understand the importance of thoroughly examining the stability and reliability of our results. In response to your valuable input, we conducted marginal analyses as a sensitivity analysis to complement our main findings. Marginal analyses involve testing the relationship between variables while controlling for additional factors or covariates that may influence the results. By conducting these additional analyses, we can assess the robustness of our findings and determine if they hold true even when considering other relevant variables.

The inclusion of marginal analyses will strengthen the validity and reliability of our study, providing a more comprehensive assessment of the relationships and outcomes examined. This approach will allow us to further evaluate the robustness of our main findings and enhance the overall quality of our research.

7. The methodology section needs to be revised to provide a clearer description of the statistical methods used, including equations and any assumptions made.

Response:

We are grateful for the feedback we received on the methodology section of our study, especially regarding the clarity of the statistical methods used. We understand the importance of providing a detailed and transparent methodology to enable other researchers to replicate our study. In response to this feedback, we have made significant revisions to the methodology section of our study. We have provided a clearer and more detailed explanation of the statistical methods employed, including relevant equations and assumptions made during the analysis. Furthermore, we have made the do files and data used, along with their sources, available in a repository for replication purposes. Our aim is to enhance the clarity and transparency of our methodology, allowing readers to better understand and replicate our study, and assess the robustness of our findings.

8. The methodology section needs to be revised to provide a clearer description of the statistical methods used, including equations and any assumptions made.

Response:

We are grateful for the feedback we received on the methodology section of our study, especially regarding the clarity of the statistical methods used. We understand the importance of providing a detailed and transparent methodology to enable other researchers to replicate our study. In response to this feedback, we have made significant revisions to the methodology section of our study. We have provided a clearer and more detailed explanation of the statistical methods employed, including relevant equations and assumptions made during the analysis. Furthermore, we have made the do files and data used, along with their sources, available in a repository for replication purposes. Our aim is to enhance the clarity and transparency of our methodology, allowing readers to better understand and replicate our study, and assess the robustness of our findings.

9. The authors should provide a more detailed discussion of the implications of their findings for policymakers, particularly as they relate to the Sustainable Development Goals.

Response:

Thank you for your feedback. In the new work, the authors have addressed the issue of providing a more detailed discussion of the implications of their findings for policymakers, particularly as they relate to the Sustainable Development Goals. They have included a comprehensive section in the discussion that outlines the policy implications of their findings and how they align with the SDGs. The authors have also provided recommendations for policymakers to consider in order to achieve the SDGs. Overall, the new work provides a more thorough analysis of the policy implications of the findings and their relevance to the SDGs.

10. Overall, the paper's concept is unique; nevertheless, non-academic language should be avoided in some cases, and in-text references should be proofed.

We appreciate the feedback provided on our paper's concept and language usage. We agree that it is essential to maintain academic language standards in scientific writing. We have taken the necessary steps to ensure that our paper adheres to these standards and have avoided non-academic language where possible. Additionally, we have reviewed and proofread our in-text references to ensure accuracy and consistency. Our aim is to produce a high-quality paper that meets the academic standards and expectations of our readers.
