# Supplementary material for: The dynamics of carbon emissions, energy, income, and life expectancy: Regional comparative analysis
Source: PLoS One. 2024 Feb 22;19(2):e0293451. doi: 10.1371/journal.pone.0293451 (PMC10883542; doi:10.1371/journal.pone.0293451)
Supplement: Attachment — Submitted filename: Response to reviewers.docx [file pone.0293451.s005.docx]

# Response to reviewers

Reviewer #3: The authors improve the quality of the paper but the "Introduction, and literature review" section still needs significant changes. The author fails to effectively present the contribution of the study, nor clearly mention the literature gaps of this study from existing literature. Again, policy recommendations need to be redesign alongside the SDGs.

# Response

We sincerely appreciate your careful review of our paper and your valuable feedback. We are pleased to inform you that we have taken your suggestions into serious consideration and have made substantial revisions to the "Introduction and Literature Review" section of the manuscript.

In response to your concerns, we have worked diligently to enhance the clarity and presentation of the paper's contribution. In the revised section, we have provided a more focused and concise explanation of the study's contribution to the existing literature. We have also taken care to highlight the specific gaps that our study addresses within the context of the existing research landscape. By doing so, we believe the introduction now more effectively communicates the significance of our work.

Furthermore, we have taken your advice to heart regarding the policy recommendations. We have carefully reconsidered and redesigned the policy recommendations section to align more closely with the Sustainable Development Goals (SDGs). This has allowed us to provide a stronger and more actionable set of recommendations that are grounded in the broader framework of sustainable development.

We hope that these revisions address your concerns and greatly enhance the overall quality of the paper. We are genuinely grateful for your constructive feedback, which has undoubtedly played a crucial role in strengthening our manuscript.
